# Pharmacological Treatments for Patients with Treatment-Resistant Depression

**DOI:** 10.3390/ph13060116

**Published:** 2020-06-04

**Authors:** Valerie L. Ruberto, Manish K. Jha, James W. Murrough

**Affiliations:** Depression and Anxiety Center for Discovery and Treatment, Icahn School of Medicine at Mount Sinai, New York, NY 10029, USA; valerie.l.ruberto@gmail.com (V.L.R.); manish.jha@mssm.edu (M.K.J.)

**Keywords:** depression, major depressive disorder, treatment resistant, antidepressant, pharmacotherapy, lithium, ketamine

## Abstract

Over a third of patients with major depressive disorder (MDD) do not have an adequate response to first-line antidepressant treatments, i.e., they have treatment-resistant depression (TRD). These patients tend to have a more severe course of illness and are at an increased risk of suicide. Next step treatment options for patients with TRD, include switching to a different antidepressant, combining more than one antidepressant, or augmenting an antidepressant with another (non-antidepressant) medication. It is unclear which of these treatment approaches should be applied to a given patient, and in what order. Due to this ambiguity, comparing antidepressants and augmentation agents on the basis of their efficacy, tolerability, and speed of symptom relief would be beneficial for clinicians. To accomplish this, a systematic search was conducted following PRISMA guidelines. Only randomized controlled trials were included in this qualitative synthesis, resulting in 66 articles. This review identified several effective pharmaco-therapeutic strategies that are currently available for patients with TRD. Ketamine and esketamine appear to be effective for the treatment of TRD. Augmentation with certain second generation antipsychotics, such as quetiapine or aripiprazole is likewise effective, and may be preferred over switching to antidepressant monotherapy. While the combination of olanzapine and fluoxetine was one of the first pharmacotherapy approved for TRD, and its use may be limited by metabolic side-effects. Other effective strategies include augmentation with lithium, liothyronine (T3), lamotrigine, or combination of antidepressants including bupropion, tricyclics, or mirtazapine. There is insufficient research to demonstrate the efficacy of ziprasidone or levothyroxine (T4). A shared decision-making approach is recommended to guide treatment selection to address each patient’s individual needs.

## 1. Introduction

Depression is a common and disabling mental disorder [1]. It is estimated that up to one in five adults in America meet the criteria for major depressive disorder (MDD) during their lifetime [1]. Unfortunately up to 70% of people will continue experiencing burdensome symptoms despite receiving evidence-based antidepressant treatment [2,3]. While there is no universally agreed upon definition of treatment-resistant depression (TRD), inadequate response to even one adequate trial of an antidepressant is a poor prognostic indicator, and inadequate response to two or more treatments of adequate dose and duration in the current major depressive episode is often used to operationalize the definition of TRD in clinical trials [4]. Depression symptoms, experienced by those who have TRD, predict a poorer outcome [5]. If symptoms do not remit, patients have an increased risk of experiencing functional impairment and committing suicide [6]. For this reason, it is imperative to identify effective treatments for individual patients.

After an inadequate response to an antidepressant, possible next step options include optimizing the dose of the current antidepressant, switching to a different antidepressant, combining antidepressants, or augmenting with a non-antidepressant medication. Each of these options have their own set of potential benefits and drawbacks. Combining antidepressants and augmenting with other medications both have the benefit of building upon a partial response to the initial antidepressant. However, adding a second medication may lead to greater side effects. On the other hand, switching from one antidepressant to another may minimize the risk of side effects, but lead to the loss of a partial therapeutic benefit to the initial antidepressant and may result in discontinuation syndrome if stopped abruptly [7,8].

A variety of antidepressant (Table 1) and augmentation (Table 2) agents have been investigated for their efficacy and safety in patients with MDD. However, their comparative efficacy in patients with TRD is not well-established. Comparing antidepressants and augmentation strategies on the basis of efficacy, tolerability, and speed of symptom relief would be informative for clinicians to guide decision making for patients with TRD.

## 2. Results

### 2.1. Review of Studies Using Antidepressant Medications

#### 2.1.1. Switch to Another Antidepressant

Patients who fail to respond to one antidepressant may respond when switched to an alternative antidepressant. Table 3 lists the antidepressant studies included in this review. This was tested in the level 2 of Sequenced Treatment Alternatives to Relieve Depression (STAR*D) Study, which compared switching to bupropion (*n* = 239), sertraline (*n* = 238), or venlafaxine (*n* = 250) in order to determine which was the most effective option in patients with MDD who had improved inadequately with citalopram and agreed to switch. Response rates to bupropion, sertraline, and venlafaxine were 26.1%, 26.7%, and 28.2%, respectively and remission rates were 21.3%, 17.6%, and 24.8%, respectively (no significant difference, *X*^2^ = 3.649, *df* = 2, *p* = 0.16). Furthermore, these three treatment arms did not differ significantly in either time to remission (*X*^2^ = 0.38, *p* = 0.93) or time to response (*X*^2^ = 0.65, *p* = 0.72). There was no significant difference in tolerability between switching to bupropion, sertraline, or venlafaxine [11].

In a separate study, Thase et al. compared switching to imipramine after failing to respond to sertraline, to switching to sertraline after failing to respond to imipramine. This study found that 55% of 117 participants who failed to respond to sertraline had a response to imipramine, and 63% of 51 patients who failed to respond to imipramine had a response to sertraline (*X*^2^ = 1.96, *p* = 0.16). The remission rates were also not significantly different, with 35% of the sertraline group remitting compared to 30% of the imipramine group (*X*^2^ = 0.94, *p* = 0.33) [12].

In level 3 of STAR*D, Fava et al. compared switching to mirtazapine monotherapy (*n* = 114) to nortriptyline monotherapy (*n* = 121) in a sample of 235 participants who failed to respond to citalopram and either an augmentation with another medication or a switch to a different antidepressant. They found no significant difference in response rates, which were 13.4% and 16.5% respectively (*p* = 0.57), remission rates, which were 8%, and 12.4%, respectively (*p* = 0.45), or tolerability between these medications [13]. Fang et al. found no significant difference in remission rates between patients who switched to mirtazapine (36.4% of 55 patients), venlafaxine (42% of 50 patients), or paroxetine (46.7% of 45 patients) in a sample of 150 participants who failed to respond to two antidepressant (*X*^2^ = 1.097, *df* = 2, *p* = 0.578) [14].

#### 2.1.2. Combining Antidepressants vs. Antidepressant Monotherapy

In the VA Augmentation and Switching Treatments for Improving Depression Outcomes (VAST-D) comparative efficacy study, Mohamed et al. compared three treatment options under randomized double blind open-label conditions in a sample of 1522 adult Veterans who failed to respond to at least one antidepressant: Switch to bupropion, augmentation with bupropion, or augmentation with aripiprazole. The study found that response to bupropion in combination with another antidepressant (65.6% of 506 patients) was not significantly more effective than response to switching to bupropion after failing an antidepressant (62.4% of 511 patients; RR, 1.05 [95% CI, 0.96–1.15]; *p* = 0.29). The same was true for remission rates, which were 26.9% and 22.3% respectively (RR, 1.20 [95% CI, 0.97–1.50]; *p* = 0.09) [15]. The results concerning aripiprazole are presented below.

Lam et al. compared bupropion monotherapy (*n* = 17), citalopram monotherapy (*n* = 12), and bupropion combined with citalopram (*n* = 32) in a sample of 61 patients that had failed either bupropion or citalopram. The combination group had a significantly higher remission rate than the monotherapy groups, 28%, and 7%, respectively (*p* < 0.05) [16]. In level 2 of the STAR*D study, Trivedi et al. compared combining bupropion with citalopram (*n* = 279) to augmenting citalopram with buspirone (*n* = 286) in a sample of 565 patients that failed to respond to citalopram. Response rates were 31.8% and 26.9% respectively (*p* = 0.21), and remission rates were 29.7% and 30.1% (X^2^ = 0.01, *p* = 0.93) [17]. Adverse events included headaches and insomnia, although these tended to be mild to moderate in severity. Combining bupropion with other antidepressants does not appear to significantly increase frequency or severity of side effects [16].

A double-blind study by Nelson et al. compared the combination of fluoxetine and desipramine (*n* = 13) to fluoxetine (*n* = 14) or desipramine monotherapy (*n* = 12) in a sample of 39 patients that had failed to respond to at least one antidepressant. This study found that the combination of fluoxetine and desipramine was more effective than fluoxetine or desipramine monotherapy. Response rates were 7.7%, 35.7%, and 16.7% respectively, and remission rates were 53.8%, 7.1%, and 0% (*X*^2^(6) = 24.01, *p* = 0.0005). Notably, symptom reduction began in as little as two weeks in that trial [18].

In level 4 of STAR*D, the combination of mirtazapine and venlafaxine (*n* = 51) was compared to tranylcypromine monotherapy (*n* = 58) in a sample of 109 patients who had previously failed three courses of antidepressant treatments. There was no significant difference in remission rates (13.7% and 6.9%), time to respond or remit, or tolerability. The response rates were 23.5% and 12.1% [19]. An open-label study by Navarro et al. compared the combination of mirtazapine and venlafaxine (*n* = 56) to imipramine monotherapy (*n* = 56) in a sample of 112 patients that failed to respond to venlafaxine. They found that imipramine monotherapy was significantly more effective. The remission rate for the combination of mirtazapine and venlafaxine was 39.28% and 71.43% for the imipramine group (X^2^ = 11.71, *p* = 0.001) [20].

A double-blind study by Carpenter et al. found that adding mirtazapine to an ongoing antidepressant (*n* = 11) resulted in significantly lower HAM-D scores than adding a placebo (*n* = 15) in a sample of 26 patients that failed to respond to one antidepressant. HAM-D scores at the endpoint were 10.7 (SD = 7.0), and 17.3 (SD = 8.2; *p* = 0.017), respectively. Remission rates were 45.5% and 13.3% (*p* = 0.068) and response rates were 63.6%, and 20%, respectively (*p* = 0.043) [21]. However, a phase III study by Kessler et al. found that adding mirtazapine (*n* = 241) did not result in significantly higher response or remission rates than adding placebo (*n* = 239) in a sample of 480 patients that failed to respond to either a selective serotonin reuptake inhibitor (SSRI) or serotonin-norepinephrine reuptake inhibitor (SNRI). The response rates for mirtazapine and the placebo were 44% and 36% respectively (*p* = 0.10), and the remission rates were 29% and 24% (*p* = 0.27). There was no significant difference in tolerability between the two arms [22].

The comparative efficacy of augmentation strategies versus switching to or combining with other antidepressants are discussed in subsequent sections.

### 2.2. Review of SGA Augmentation Studies

Second generation antipsychotics (SGA) such as aripiprazole, olanzapine/fluoxetine combination, quetiapine, brexpiprazole, risperidone, and ziprasidone, are effective when used as augmentation agents in adults with MDD who fail to respond to a monoaminergic antidepressant. Table 4 lists the SGA studies included in this review.

Aripiprazole is approved by the Food and Drug Administration (FDA) as an augmentation agent for MDD in doses ranging from 2 mg/day to 15 mg/day. The available data suggest that patients can respond to this augmentation treatment in as little as two weeks [27]. A rater-blind study by Han et al. compared augmenting an antidepressant with aripiprazole (*n* = 52) to switching to a different antidepressant (*n* = 49) in a sample of 101 participants who had failed to respond to one antidepressant in the current depressive episode. Participants in the aripiprazole group received a starting dose of 2 or 5 mg/day and the dose was increased by 2–5 mg/day at each visit, to a maximum dose of 15 mg/day. Augmenting with aripiprazole was significantly more effective than switching to a different antidepressant. Response rates were 60%, and 32.6%, respectively (*p* = 0.0086), and remission rates were 54% and 19.6% (*p* = 0.0005) [28].

A double-blind study by Marcus et al. compared aripiprazole augmentation (*n* = 191) to placebo augmentation of an antidepressant (*n* = 190) in a sample of 381 participants who failed to respond to an antidepressant in the current depressive episode. Aripiprazole augmentation resulted in significantly higher remission (25.4% vs. 15.2%; *p* = 0.016) and response rates (32.4% vs. 17.4%; *p* < 0.001) than the placebo augmentation. The mean dose at the endpoint of this study was 11.0 mg/day, with participants receiving anywhere from 2 to 20 mg/day [27]. Similarly, a double-blind study by Berman et al. showed that aripiprazole augmentation of an antidepressant (*n* = 177) led to significantly greater response (46.6% vs. 26.6%; *p* < 0.001) and remission rates (36.8% vs. 18.9%; *p* < 0.001) compared to a placebo augmentation (*n* = 172) in a sample of 349 patients who failed to respond to an antidepressant in the current depressive episode. The mean dose at the endpoint of this study was 13.9 mg/day, with participants taking between 2 and 20 mg/day [29].

In the VAST-D study mentioned earlier that compared switching to bupropion (*n* = 511), augmentation with bupropion (*n* = 506), and augmentation with aripiprazole (*n* = 505), augmentation with aripiprazole resulted in significantly higher response rates than combining bupropion with another antidepressant (74.3% vs. 65.6%; RR, 1.13 [95% CI, 1.04–1.23]; *p* = 0.003), as well as switching to bupropion (74.3% vs. 62.4%; RR, 1.19 [95% CI, 1.09–1.29]; *p* < 0.001). The remission rate for the aripiprazole group was significantly higher than the group that switched to bupropion (28.9% vs. 22.3%; RR, 1.03 [95% CI, 1.05–1.60]; *p* = 0.02), but did not significantly differ from the group that received a combination of bupropion and another antidepressant (28.9% vs. 26.9%; RR, 1.08 [95% CI, 0.88–1.31]; *p* = 0.47) [15].

Yoshimura et al. compared aripiprazole augmentation of paroxetine to aripiprazole augmentation of sertraline in a sample of 26 patients that failed to respond to two antidepressants from different drug classes. There was no significant difference in response rates (27.7% vs. 15.4%; *p* = 0.475) between the two groups [30]. Taken together, these findings suggest that aripiprazole is an effective augmentation agent regardless of the background antidepressant.

However, one study shows different results regarding the efficacy of aripiprazole. A double-blind study by Fava et al. showed no significant difference in response or remission rates between a group of participants receiving 2 mg/day of aripiprazole (*n* = 56) and a group receiving placebo augmentation (*n* = 169) in a sample of 225 participants who failed to respond to an antidepressant. The response rates were 18.5% for the group receiving aripiprazole and 17.4% for the group receiving the placebo, with a weighted difference of 5.62% ([95% CI, −2.69–13.94], *p* = 0.18). The remission rates were 7.41% and 9.58% respectively, with a weighted difference of 2.30% ([95% CI, −4.35–8.94], *p* = 0.49) [31]. This may suggest that low doses of aripiprazole are not as effective as higher ones.

Akathisia is the most common adverse event experienced with aripiprazole (25%, vs. 4% with placebo), occurring at doses as low as 2 mg/day. Some MDD patients who take aripiprazole also experience restlessness (12%, vs. 2% with placebo), insomnia (8%, vs. 2% with placebo), fatigue (8%, vs. 4% with placebo), and blurred vision (5%, vs. 1% with placebo) [32]. Higher doses of aripiprazole result in more adverse events than lower doses do [31]. Overall, augmenting antidepressants with aripiprazole in doses between 2 mg and 20 mg/day is relatively well-tolerated [29].

A combination product of olanzapine and fluoxetine was the first medication approved by the U.S. FDA for adults with TRD. Here, the term “combination” is used to describe the simultaneous co-administration of two medications; more often the term “augmentation” is used to describe the use of an antidepressant concurrently with a second, non-antidepressant medication (e.g., an SGA), while “combination” often refers to co-administration of two separate antidepressants. Shelton et al. compared this olanzapine-fluoxetine combination (OFC; *n* = 9) to olanzapine monotherapy (*n* = 6) and fluoxetine monotherapy (*n* = 9) in a sample of 24 patients who failed to respond to at least one antidepressant. The response rate for the combination group (60%) was significantly higher than the olanzapine monotherapy group (0%; *p* = 0.03), but not the fluoxetine monotherapy group (10%; *p* = 0.11). However, the combination group had a significantly greater improvement from baseline on the Montgomery-Asberg Depression Rating Scale (MADRS) than both the olanzapine monotherapy group (−13.6 vs. −2.8; pair-wise F = 2.22, df = 8, 176, *p* = 0.03) and the fluoxetine monotherapy group (−13.6 vs. −1.2; pair-wise F = 2.78, df = 8, 176, *p* = 0.006) [33]. Similarly, a double-blind study by Thase et al. compared OFC (*n* = 200) to olanzapine (*n* = 199) and fluoxetine monotherapy (*n* = 206) in a sample that failed fluoxetine and one other antidepressant in the current depressive episode. OFC significantly decreased MADRS total score (−14.5) compared to olanzapine (−7.0; *p* < 0.001) and fluoxetine monotherapy (−8.6; *p* < 0.001). Remission rates were 27% for the OFC group, 17% for the fluoxetine monotherapy group, and 15% for the olanzapine monotherapy group [34].

However, another study by Shelton et al. showed conflicting results. This study compared OFC (*n* = 146) to olanzapine (*n* = 144), fluoxetine (*n* = 142), and nortriptyline monotherapy (*n* = 68) in a sample of 500 patients that failed to respond to at least one SSRI. The OFC group only had a significantly greater decrease in MADRS scores than the olanzapine group at the endpoint of the study (*p* = 0.005) [35]. A double-blind study by Corya et al. also raised questions about the comparative efficacy of OFC in a sample that failed to respond to an SSRI and venlafaxine. The treatment arms in this study were as follows: OFC (*n* = 243), olanzapine monotherapy (*n* = 62), fluoxetine monotherapy (*n* = 60), venlafaxine monotherapy (*n* = 59), and a lower dose of OFC containing 1 mg/day of olanzapine and 5 mg/day of fluoxetine, rather than the typical 6 mg/day of olanzapine and 25 mg/day of fluoxetine dose (*n* = 59). The response rate of the OFC group (43.3%) was only significantly higher than the olanzapine monotherapy group (25.4%; *p* = 0.017), but was not significantly different than the fluoxetine monotherapy (33.9%), venlafaxine (50.0%), or low dose OFC groups (36.4%). Similarly, the remission rate of the OFC group (29.9%) was only significantly higher than the olanzapine monotherapy group (13.6%; *p* = 0.013), but not significantly different than the fluoxetine monotherapy (17.9%), venlafaxine (22.4%), or low dose OFC groups (20.0%) [36].

OFC can cause metabolic changes, such as hyperglycemia, dyslipidemia, and weight gain. After 12 weeks of exposure to OFC, 34.1% of people had a random glucose reading of 140 mg/dl or higher, compared to 3.6% of people taking a placebo. 36.2% of people taking OFC had a non-fasting total cholesterol of 200 mg/dl or higher, compared to 9.9% of people taking a placebo. These metabolic changes may increase a patient’s risk of cardiovascular and cerebrovascular side effects [37].

Quetiapine is another SGA that has evidence of efficacy in depression symptoms in patients who have shown non-response to prior antidepressant treatments. It is also approved by the FDA as an augmentation agent for the treatment of depression. In some cases, quetiapine separates from placebo within one week of treatment [38,39]. It is administered in doses of 150 mg/day or 300 mg/day. A placebo-controlled study by Cutler et al. compared a 150 mg/day (*n* = 152) and a 300 mg/day dose of quetiapine augmentation of an antidepressant (*n* = 152) to placebo augmentation (*n* = 157). Response rates for the 150 mg/day (54.4%; *p* < 0.01) and the 300 mg/day groups (55.1%; *p* < 0.01) were both significantly more effective than the placebo (36.2%) in a sample that had failed to respond to fewer than two antidepressants in the current depressive episode. However, only the remission rate of the 300 mg/day group (32.0%; *p* < 0.05) was significantly more effective than the placebo (20.4%; *p* < 0.05). The remission rate for the 150 mg/day group was 26.5% (*p* = 0.267) [38]. A double-blind study by El-Khalili et al. showed similar results in a sample of 446 patients that failed to respond to at least one antidepressant in the current depressive episode. A 300 mg/day dose of quetiapine (*n* = 150) was significantly more effective than the placebo (*n* = 148), with a response rate of 58.9%, compared to 46.2% for the placebo (*p* < 0.05), and a remission rate of 42.5%, compared to 24.5% for the placebo (*p* < 0.01). The 150 mg/day dose (*n* = 148) did not have a significantly different response rate than the placebo (51.7% vs. 46.2%; *p* = 0.329), but there was a trend towards a higher remission rate (35.0% vs. 24.5%; *p* = 0.059) [39]. A double-blind study by Bauer et al. compared a 300 mg/day dose (*n* = 163), a 150 mg/day dose (*n* = 167), and a placebo (*n* = 163) in a sample of 493 patients that failed to respond to at least one antidepressant. The response rate for the 300 mg/day group was 57.8% (*p* < 0.05 vs. placebo), the 150 mg/day group was 55.4% (*p* = 0.107 vs. placebo), and the placebo group was 46.3%. However, remission rates were 31.1% (*p* = 0.126 vs. placebo), 36.1% (*p* < 0.05 vs. placebo), 23.8% respectively [40].

However, a study by Hobart et al. found that quetiapine augmentation of an antidepressant (*n* = 100) did not separate from the placebo augmentation of an antidepressant (*n* = 206) in a sample of patients who had failed to respond to between one and three antidepressants. The response rates were 8.1% for the quetiapine group and 6.8% for the placebo group (*p* = 0.60). The remission rates were 2.0% for the quetiapine group and 4.4% for the placebo group (*p* = 0.39) [41].

As with OFC, quetiapine can cause metabolic disturbances, including hyperglycemia, dyslipidemia, and weight gain. 12% of MDD patients taking a 300 mg/day dose of quetiapine shifted from a normal baseline blood glucose to 126 mg/dl or higher. This rate was only 7% for those taking a 150 mg/day dose, compared to 6% for people taking a placebo. While, 16% of MDD patients taking quetiapine shifted from normal total cholesterol levels to a clinically significant value of 240 mg/dl or higher, compared to 7% of those taking a placebo. Of the MDD patients taking quetiapine, 5% gained at least 7% of their body weight, compared to only 2% of those taking a placebo [42].

Brexpiprazole is also FDA approved as an augmentation agent for the treatment of depression. A phase III study by Thase et al. found that a 2mg/day dose of brexpiprazole (*n* = 175) was significantly more effective than the placebo (*n* = 178) in a sample of patients who have had an inadequate response to at least one antidepressant. While, 23.4% of the brexpiprazole group responded, compared to 15.7% in the placebo group (LS mean = 1.54 [95% CI, 1.01–2.35]; *p* = 0.0429), and 14.9% of the brexpiprazole group remitted, compared to 9.0% of the placebo group (LS mean = 1.67 [95% CI, 0.97–2.90]; *p* = 0.0671). Although, the remission rate was not significant, brexpiprazole decreased MADRS total scores by an average of -8.36 compared to the placebo, which caused an average decrease of −5.15 (LS mean = −3.21 [95% CI, −4.87–−1.54]; *p* = 0.0002) [43].

Thase et al. reported on another phase 3 study comparing a 1 mg/day (*n* = 211) and a 3mg/day dose (*n* = 213) in a sample of patients that has failed between one and three antidepressants. Similar to the 2 mg/day dose, the 1 mg/day and 3 mg/day doses had response rates that were significantly higher than the placebo (*n* = 203), but remission rates that were not. Response rates were 14.3% for the placebo group, 23.2% for the 1 mg/day group (LS mean = 1.69 [95% CI, 1.14–2.50]; *p* = 0.0094), and 23.0% for the 3 mg/day group (LS mean = 1.65 [95% CI, 1.09–2.50]; *p* = 0.0162). The average decrease in MADRS score from baseline to endpoint was −8.29 for the 3 mg/day group compared to the placebo group (−6.33; LS mean = −1.95 [95% CI, −3.39–−0.51]; *p* = 0.0079), and −7.64 for the 1 mg/day group (LS mean = −1.30 [95% CI, −2.73–0.13]; *p* = 0.0737). The 1 mg/day dose was not more effective than the placebo, but the 3 mg/day dose was. Brexpiprazole may be effective in as little as one week [44].

Hobart et al. found that brexpiprazole augmentation of an antidepressant (*n* = 197) was not associated with significantly higher response (10.5% vs. 6.8%; *p* = 0.22) or remission rates (6.8% vs. 4.4%; *p* = 0.33) when compared to an adjunctive placebo (*n* = 206) in a sample of patients that had failed to respond to between one and three antidepressants in the current depressive episode. However, brexpiprazole did result in significantly greater change in MADRS score from baseline to endpoint than the placebo, with the brexpiprazole group experiencing a mean change of -6 compared to −4.6 in the placebo group (LS mean = −1.48 [95% CI, −2.56–−0.39]; *p* = 0.0078). This study used a 2-3 mg/day dose of brexpiprazole [41]. The most frequent adverse events are weight gain (7%, vs. 2% with placebo) and akathisia (9%, vs. 2% with placebo). Akathisia appears to be dose dependent [45].

Risperidone has shown positive results as an augmentation agent in patients with unipolar depression in some studies. However, it is not FDA approved as an augmentation agent for depression [46]. Mahmoud et al. found that more participants receiving risperidone (*n* = 137) experienced response than participants receiving a placebo (*n* = 131) in a sample of participants who failed to respond to one antidepressant in the current episode (46.2% vs. 29.5%; *p* = 0.004). The same was true for remission rates (24.5% vs. 10.7%; *p* = 0.004) [47]. Keitner et al. found similar results in a sample of 94 participants. 54.8% of the 64 patients in the risperidone group responded, compared to 33.3% of the 30 patients in the placebo group (CMH = 3.88, df = 1, *p* = 0.049) and 51.6% of the risperidone group remitted, compared to 24.2% of the placebo group (CMH = 6.48, df = 1, *p* = 0.011) [48].

A double-blind study by Reeves et al. found that risperidone (*n* = 12) is significantly more effective than placebo (*n* = 11) in a sample of patients that failed to respond to two antidepressants. The mean change in MADRS score at week 6 was −21.68 (SE = 2.28) for the risperidone group and −11.39 (SE = 2.53) for the placebo group (p = 0.0087) [46]. A pilot study by Fang et al. compared the efficacy of risperidone (*n* = 45), thyroid hormone (*n* = 48), buspirone (*n* = 46), valproate (*n* = 39), and trazadone augmentation of an antidepressant (*n* = 47) in a sample of 225 participants who failed at least one antidepressant. There was no significant difference in efficacy or adverse events between these treatment arms. Response rates were 46.7% for the risperidone group, 61.5% for the valproate group, 56.5% for the buspirone group, 61.7% for the trazodone group, and 58.3% for the thyroid hormone group (F/X^2^ = 2.748, *p* = 0.601). Remission rates were 26.7%, 48.7%, 32.6%, 42.6%, and 37.5% respectively (F/X^2^ = 5.336, *p* = 0.255) [49]. Risperidone is generally well-tolerated. Keitner et al. found participants receiving risperidone, and participants receiving a placebo, reported the same number of adverse events [47]. Some participants experience dry mouth, headache, and somnolence [48].

Ziprasidone is another SGA that has demonstrated some efficacy for TRD, but is not FDA approved for this indication. A double-blind study by Papakostas et al. compared augmentation of escitalopram with ziprasidone (*n* = 71) to escitalopram with a placebo (*n* = 68) in a sample of 139 participants who failed to respond to escitalopram. The response rate for the ziprasidone group (35.2%) was significantly higher than the placebo group (20.5%; *p* = 0.04). However, the remission rate for the ziprasidone group (38.0%) was not significantly different than the placebo group (30.8%; *p* = 0.32) [50]. In another double-blind study by Papakostas et al., ziprasidone monotherapy (*n* = 21) was compared to a placebo (*n* = 25) in a sample of 120 patients. Response rates (5% vs. 7%; *p* = 0.59) and remission rates (7% vs. 10%; *p* = 0.73) did not differ significantly [51].

### 2.3. Review of Lithium Augementation Studies

Lithium has shown efficacy as an augmentation agent for TRD in several controlled trials, although it is not FDA approved for this indication. Table 5 lists the lithium studies included in this review. Heninger et al. compared lithium augmentation of an antidepressant (*n* = 8) to placebo augmentation (*n* = 7) in a sample of 15 patients who failed to respond to at least one antidepressant. They found that the lithium group experienced a decrease in scores on the global depression item of the Short Clinical Rating Scale (SCRS) from 6.1 to 3.3 (*p* < 0.012), compared to an increase from 5.6 to 5.8 in the placebo group. Lithium separated from placebo in as little as one week [55]. A double-blind study by Schöpf et al. found similar results in a sample of 27 patients that failed to respond to a tricyclic antidepressant (TCA). The group receiving lithium augmentation (*n* = 14) experienced a decrease in Hamilton Depression Rating Scale (HAM-D) scores from 19.3 (SD = 4.9) to 10.4 (SD = 5.3) in one week (*p* < 0.001). The placebo group (*n* = 13) remained more or less unchanged, starting at 20.4 (SD = 4.3), and decreasing to 19.3 (SD = 5.8) [56].

Katona et al. found that lithium (*n* = 29) was significantly more effective than placebo (*n* = 32) in a sample of patients that had failed to respond to fluoxetine or lofepramine. Response rates were 52% for the lithium augmentation group and 25% for the placebo group (X^2^ = 4.6, *p* < 0.05) [57]. Cappiello et al. found that significantly more people responded to lithium augmentation compared to placebo in a sample of 31 patients. 28.6% of the 14 patients in the lithium group responded compared to 0% of the 15 patients in the placebo group (*p* < 0.042) [58]. Zusky et al. found no difference between 300 mg/day of lithium (*n* = 8) and placebo (*n* = 8) in a sample of treatment-resistant participants, however, the small sample size may have led to type II errors [59]. Low-dose lithium is not effective. Stein et al. found that 750 mg/day of lithium was significantly more effective than placebo, but 250 mg/day was not in a sample of 34 TCA-resistant participants. Response rates were 44% for patients taking 750 mg/day of lithium, compared to 18% of patients taking 250 mg/day of lithium (X^2^ = 14.45, df = 1, *p* < 0.001). 22% responded to placebo [60].

Lithium is no more effective than antidepressant monotherapy, according to a double-blind study by Fava et al. This study compared high doses of fluoxetine (40–60 mg/day; *n* = 15), fluoxetine (20 mg/day) augmented with lithium (*n* = 14), and a combination of fluoxetine (20 mg/day) and desipramine (*n* = 12) in a sample of 41 participants who failed to respond to a 20 mg/day dose of fluoxetine. The response rates were 29% for the lithium plus fluoxetine group, 25% for the desipramine plus fluoxetine group, and 53% for the high-dose fluoxetine group (X^2^ = 2.9, df = 2, *p* = 0.24). Although the response rates did not differ significantly between these groups, the mean change in HAM-D total score from baseline to endpoint did. The lithium group experienced a mean change of 9.5 (SD = 2.7), the desipramine group experienced a change of 3.4 (SD = 4.8), and the high dose fluoxetine group experienced a change of 9.0 (SD = 5.3; *p* = 0.04). High-dose fluoxetine, and lithium plus fluoxetine, were significantly more effective than desipramine plus fluoxetine [25]. Fava et al. conducted a similar double-blind study with a sample size of 101 participants in 2002. The response rates were 23.5% of 34 patients in the lithium plus fluoxetine group, 29.4% of 34 patients in the desipramine plus fluoxetine group, and 42.4% of 33 patients in the high dose fluoxetine group (X^2^ = 2.9, *p* = 0.2) [26]. Nierenberg et al. compared lithium augmentation of nortriptyline (*n* = 18) to a placebo (*n* = 17) in a sample of 35 participants who had failed to respond to between one and five antidepressants in the current depressive episode. 11.1% of the lithium group responded, compared to 17.6% of the placebo group (p = NS). There was no significant difference in efficacy of these treatments [61].

Lithium augmentation may have similar efficacy in TRD compared to augmentation with an SGA. A study by Yoshimura et al. compared augmentation of paroxetine with lithium (*n* = 10), olanzapine (*n* = 10), and aripiprazole (*n* = 10) in a sample of 30 participants who had previously failed paroxetine. The response rates were 40% for the lithium group, 30% for the olanzapine group, and 40% for the aripiprazole group. Remission rates were 20%, 10%, and 20%, respectively. The significance of these results is not stated, however, the change in HAM-D score from baseline to endpoint was significant for all groups (*p* < 0.001) [52]. Bauer et al. showed similar results when comparing lithium augmentation (*n* = 229) to quetiapine augmentation (*n* = 231), and quetiapine monotherapy (*n* = 228) in a sample of 688 patients who had an inadequate response to at least one antidepressant. The group receiving adjunctive quetiapine had a response rate of 52.4%, the group receiving quetiapine monotherapy had a response rate of 50.7%, and the group receiving adjunctive lithium had a response rate of 46.2% (*p* = 0.6912). Remission rates were 31.9%, 23.6%, and 27.1%, respectively (*p* = 0.6496) [54]. Adverse events include tremors, headache, nausea, and akathisia [54].

### 2.4. Review of Thyroid Hormone Augmentation Studies

The use of the thyroid hormones, T3 and T4, in patients with TRD has been researched for decades. T3 may be an effective augmentation agent for depression, although it is not FDA approved. Table 6 lists the thyroid hormone studies included in this review. It shows similar efficacy to other augmentation agents. As part of the STAR*D study, Nierenberg et al. compared lithium (*n* = 69) to T3 (*n* = 73) in a sample of participants who failed at least two antidepressants. While, 16.2% of participants in the lithium group responded to treatment, compared to 23.3% in the T3 group (X^2^ = 1.70, df = 1, *p* = 0.1918). While, 15.9% of the lithium group and 24.7% of the T3 group achieved remission (X^2^ = 0.63, df = 1, *p* = 0.4258). There was no significant difference in efficacy between T3 and lithium, however, the group receiving T3 reported significantly fewer adverse events than the group receiving lithium (*p* = 0.045) [63]. Joffe et al. found similar results in a sample of 50 patients who had failed to respond to a TCA. However, both were significantly more effective than the placebo. A total of 41.2% of the 17 patients that received T3 responded (vs 12.5% of the 16 patients that received placebo; *p* = 0.058), 35.3% of the 17 patients that received lithium responded (*p* = 0.098). Although these results were not significant, the mean changes in HAM-D score were. The mean score of the placebo group at the endpoint was 14.9 (SD = 8.0), the T3 group was 11.0 (SD = 6.4; F = 4.86, df = 1, 32; *p* = 0.035), and the lithium group was 12.1 (SD = 7.3; F = 7.62, df = 1, 32; *p* = 0.01) [62]. In another study, Joffe et al. showed that T3 (*n* = 10), lithium (*n* = 9), and the combination of both (*n* = 9) showed no significant difference from placebo (*n* = 8) in a sample of participants who failed to respond to one antidepressant. There was no significant difference in change in HAM-D score between the groups (F = 0.551, df = 3, *p* = 0.651). Symptom relief may begin in as early as two weeks [64].

Joffe et al. investigated whether T3 (*n* = 17) is more effective than L-thyroxine (T4; *n* = 21) in a sample of 38 participants who had failed to respond to a TCA. The results showed that significantly more people responded to T3 (52.9% vs. 19.0%; *p* = 0.026). The T3 group had significantly lower HAM-D scores at the endpoint (12.6; SD = 6.2) than the T4 group (16.6; SD = 6.3; *p* < 0.05) [66]. Fang et al. found that there was no significant difference in efficacy or number of adverse events while taking thyroid hormone (*n* = 48), risperidone (*n* = 45), valproate (*n* = 39), buspirone (*n* = 46), or trazodone (*n* = 47) in a sample of 225 participants who failed at least one antidepressant. Response rates were 58.3%, 46.7%, 61.5%, 56.5%, and 61.7% respectively (F/X^2^ = 2.748, *p* = 0.601). Remission rates were 26.7%, 48.7%, 32.6%, 42.6%, and 37.5% (F/X^2^ = 5.336, *p* = 0.255) [49].

Some studies question the efficacy of T3. A double-blind study by Appelhof et al. found no significant difference between a 25 μg/d dose of T3 (*n* = 30), a 50 μg/d dose (*n* = 30), and a placebo (*n* = 53). The response rate was 46% for each group (X^2^ = 0.002, *p* = 0.99). When the two T3 groups were combined and compared with the placebo group, the difference in response rate was 0.004 (95% CI, −0.187–0.195). The remission rates were 36% in the placebo group and 32% in both T3 groups (X^2^ = 0.175, df = 2, *p* = 0.92) [67]. Gitlin et al. also found that, compared to a placebo, T3 had no significant effect on the depression symptoms of 16 patients who had failed to respond to imipramine (F = 2.68, df = 1, *p* = 0.12) [68]. T3 is well-tolerated. Some people experience palpitations, nervousness, sweating, and tremors [67]. It should not be used for patients who have hyperthyroidism.

### 2.5. Review of Lamotrigine Augmentation Studies

Many anticonvulsants have been studied for the treatment of TRD, however, studies investigating the efficacy of lamotrigine, for the most part, were the only ones that met the inclusion criteria for this review (Table 7). Lamotrigine is not an FDA approved augmentation agent for depression. There is conflicting evidence regarding the efficacy of lamotrigine for TRD. In a double-blind study by Santos et al., there was no significant difference in response rates between lamotrigine (26.7%; *n* = 17) and a placebo (35.7%; *n* = 17) in a sample of 34 participants who failed two antidepressants (*p* = 0.6). The small sample size may have impacted these results [69]. A double-blind study by Barbee et al. showed similar results in a sample of 96 patients that failed to respond to one antidepressant. The response rate was 33% out of 48 patients for both groups (*p* = 0.956) [70]. A double-blind study by Barbosa et al. found a significant difference in response rates between lamotrigine (84.62%; *n* = 13) and a placebo (30%; *n* = 10) in a sample of 23 patients that failed one antidepressant (*p* = 0.013). However, there was no significant difference in total scores on the HAM-D or MADRS [71]. A double-blind study by Normann et al. also did not find any significant difference between lamotrigine (*n* = 20) and a placebo (*n* = 20). Response rates were 55% and 50% respectively (*p* = NS) [72]. Lamotrigine may be as effective as lithium in the treatment of TRD. An open-label study by Schindler et al. found no significant difference between lamotrigine (*n* = 17) and lithium augmentation (*n* = 17) in a sample of 34 participants who failed to respond to two antidepressants. The response rates were 53% and 41% respectively, and the remission rates were 23% and 18% respectively [65]. Adverse events are not severe and include nausea, rash, and dyspepsia [69,72].

### 2.6. Review of Ketamine and Esketamine Studies

Recent research has demonstrated the antidepressant effects of ketamine. Table 8 lists the ketamine and esketamine studies included in this review. Berman et al. found that ketamine (*n* = 7) produced significantly greater reductions in HAM-D scores than saline (*n* = 7). 50% of participants responded to ketamine, compared to 12.5% who responded to the placebo (*p* > 0.05). Although the difference in response rate was not significant, there was a significant condition-by-time effect on HAM-D scores (F = 3.97, df = 5, 30; *p* = 0.02) [73]. Zarate et al. found that ketamine monotherapy (*n* = 17) was significantly more effective than a placebo (*n* = 14) in a sample of patients who had failed at least 2 antidepressants (F_1,203_ = 58.24; *p* < 0.001). 71% of patients who received ketamine responded, compared to 0% of people who received the placebo. 29% of the ketamine group remitted, compared to 0% of the placebo group. It was not specified if these results were significant or not [74]. Murrough et al. showed similar results in a sample of participants who had failed at least 3 antidepressants. This study showed that the ketamine group (*n* = 47) had significantly better response rates (64%) than the group receiving an active placebo, midazolam (*n* = 25; 28%; *p* <0.006). 24 h after treatment, the average MADRS score in the ketamine group was 14.77 (95% CI, 11.73 – 17.80), compared to 22.72 in the placebo group ([95% CI, 18.85–26.59]; t = 3.34, df = 68, *p* < 0.001) [75].

However, a double-blind study by Ionescu et al. found no significant difference in efficacy of treatment with ketamine (*n* = 13) versus saline placebo (*n* = 13) in a sample of 26 patients who had previously failed at least 5 antidepressants. 25% of patients receiving ketamine responded to treatment, compared to 33% in the placebo group (*p* > 0.05). Remission rates were 17% and 8% respectively (*p* > 0.05). 50% of participants in this study had previously failed to respond to electroconvulsive therapy (ECT). The severity of treatment-resistance in this sample may account for these insignificant results [76].

Ketamine has been researched in doses between 0.2 to 1 mg/kg, with 0.5 mg/kg being most common. In a study by Su et al., ketamine doses of 0.2 mg/kg (*n* = 23) and 0.5 mg/kg (*n* = 24) were compared with saline (*n* = 24) in a sample of 71 participants who failed more than two antidepressants. The 0.5 mg/kg dose was not significantly better than the 0.2 mg/kg dose. The response rate was 39.1% for the 0.2 mg/kg group (*p* = 0.05), 45.8% for the 0.5 mg/kg group (*p* = 0.01), and 12.5% for the placebo. However, the response rates between the two ketamine groups was not significantly different (*p* = 0.77) [77]. A double-blind study by Fava et al. found that a 0.5 mg/kg dose and a 1.0 mg/kg dose were significantly more effective than an active placebo, midazolam, in a sample of 99 patients that failed to respond to at least 2 antidepressants (*p* < 0.0001). Response rates for the 0.1 mg/kg (*n* = 18), 0.2 mg/kg (*n* = 20), 0.5 mg/kg (*n* = 22), 1.0 mg/kg (*n* = 20), and midazolam (*n* = 19) groups were 31%, 21%, 59%, 53%, and 11% respectively (*p* = 0.04224) [78]. Lenze et al. investigated whether a 96-h ketamine infusion at a dose of 0.6 mg/kg (*n* = 10) would be more effective than a 40-min dose of 0.5 mg/kg (*n* = 10) in a sample of 20 patients that failed two antidepressants in the current depressive episode. Response rates were 40% and 20% respectively. These results were not significant [79].

A double-blind study by Domany et al. investigated the use of ketamine given orally to treat depression in a sample of 41 participants who had failed to respond to at least two antidepressants. The response rate was a significantly higher in the ketamine group (*n* = 22; 31.8%) compared to the placebo group (*n* = 19; 5.6%; X^2^(1) = 4.27, *p* < 0.05). Remission rates were 27.3%, and 0% respectively (X^2^(1) = 5.78, *p* < 0.05) [80]. A double-blind crossover study by Lapidus et al. compared intranasal ketamine to a placebo in a sample of 20 patients who failed at least one antidepressant in the current depressive episode. This study found that depression symptoms were significantly improved in the ketamine condition compared to placebo 24 h after treatment (t = 4.39; *p* < 0.001). Response rates were 44% and 6% respectively (*p* = 0.033) [81]. Adverse events experienced with ketamine, include headaches, drowsiness, nausea, abnormal sensations, and acute dissociation experienced during or immediately after treatment [82]. Ketamine can also reduce blood pressure. Adverse events are temporary and mild [79]. Ketamine produces a rapid antidepressant effect [79,82]. However, these antidepressant effects may not be sustained.

A placebo-controlled study by Singh et al. compared intravenous esketamine in doses of 0.2 mg/kg (*n* = 9) and 0.4 mg/kg (*n* = 11) to a placebo (*n* = 10) in a sample of 30 people that failed one antidepressant in the current episode. Response rates were 67% (*p* = 0.013), 64% (*p* = 0.014), and 0%, respectively. The least squares mean changes from baseline to endpoint were −3.8 (SE = 2.97) for placebo, −16.8 (SE = 3.00) for the 0.2 mg/kg group, and −16.9 (SE = 2.61) for the 0.4 mg/kg group [82].

Intranasal esketamine has recently been approved by the FDA for patients with TRD. A double-blind study by Popova et al. also found that intranasal esketamine caused a significantly greater decrease in MADRS score in a sample of 39 patients that failed to respond to at least one antidepressant. Response rates for the esketamine (*n* = 114) and placebo groups (*n* = 109) were 69.3% and 52.0% respectively (odds ratio = 2.4, 95% CI = 1.30, 4.54). Remission rates were 52.5% and 31.0%, although it is not stated if these results are significant or not [83]. A double-blind study by Fedgchin et al. found that intranasal esketamine in a dose of 84 mg (*n* = 114) did not cause a significantly greater decrease in MADRS score compared to a placebo (*n* = 113) in a sample of participants who failed to respond to at least two antidepressants (*p* = 0.088). However, the LS mean difference for the group that received 56 mg of esketamine (*n* = 115) was significant (−4.1; [−7.76, −0.49], *p* = 0.027). Response rates were 54.1% for the 56 mg esketamine group, 53.1% for the 84 mg esketamine group, and 38.9% for the placebo group. Remission rates were 36.0%, 38.8%, and 30.6% respectively. It is not stated whether these results are significant or not [84]. Daly et al. found a significant dose response relationship when administering either 28 mg (*n* = 11), 56 mg (*n* = 11), or 84 mg of esketamine (*n* = 12) or a placebo (*n* = 33) to a sample of participants that failed to respond to at least two antidepressants. Higher doses were more effective, but all doses of esketamine were significantly more effective than the placebo. LS means at 24 h were −5.7 for the placebo group (SE = 1.79), −14.8 for the 28 mg esketamine group (SE = 2.80; *p* = 0.002), −15.7 for the 56 mg group (SE = 2.74; *p* < 0.001), and −16.4 for the 84 mg esketamine group (SE = 2.64; *p* < 0.001). Response rates were 36%, 27.3%, 42%, and 3% respectively. Remission rates were 36%, 18%, 25%, and 0%. It is not stated whether these results are significant or not [85]. The most frequent adverse events when using intranasal esketamine are nausea, dizziness, and dissociation [86].

One challenge when administering intranasal ketamine is that absorption can vary between individuals [87]. In addition, administration logistics can be challenging. In a study by Gálvez et al., participants had difficulty self-administering all 10 sprays of intranasal ketamine due to lack of coordination experienced as a side effect [88]. For greater dosing accuracy and ease of administration, intravenous delivery is preferred.

## 3. Discussion

The results of this review show that multiple psychopharmacological interventions have potential efficacy for adults with TRD, including antidepressants, augmentation with SGAs, lithium, T3, and more recently ketamine and esketamine. Antidepressants may be effective when combined or when switching from one antidepressant to another. SGAs seem to be effective as augmentation agents. Many are FDA approved for MDD and, for most, there are many studies that have confirmed their efficacy, particularly when compared with switching to another antidepressant [15]. However, it is important to note that metabolic changes can occur with some SGAs, especially OFC. Lithium seems to be more effective than combining a TCA with another antidepressant [25,55]. It seems to have similar efficacy to augmentation with SGAs [52,54]. T3 and lamotrigine are also effective treatments for TRD, and may be as effective as lithium [62,63,65]. Most recently, ketamine and esketamine show good efficacy data for TRD. There is insufficient evidence to determine the efficacy of ziprasidone and T4. More research must be done on these drugs before a conclusion can be made.

Many of these antidepressants and augmentation agents have similar efficacy. However, they each have a unique set of benefits and drawbacks regarding tolerability and response time. When treating TRD in a clinical setting, the best course of action is to take a shared decision-making approach to determine which factors should be prioritized for each individual patient. For some patients, especially those suffering from severe suicidal ideation, time to respond is the most important factor in choosing a medication. For these patients, it may be worth sacrificing tolerability for faster action, provided that the side effects are not severe. For acutely suicidal patients, ketamine and esketamine might be considered in particular, based on available data that suggests a rapid onset of action and activity against suicidal ideation.

It should be noted that some research has been done on the antidepressant effects of amantadine, although no studies involving amantadine fit the inclusion criteria of this review. A review by Raupp-Barcaro et al. discussed the pre-clinical and clinical evidence for the antidepressant effects of augmentation with amantadine. These studies suggest that amantadine may show promise as a treatment for TRD, however, it is too soon to know for sure. The clinical studies included in the Raupp-Barcaro review had small sample sizes and most used an open label design. Amantadine may work similarly to ketamine since both are NMDA receptor antagonists. However, amantadine may offer some advantages over ketamine, such as an oral route of administration, rather than an intravenous one. More research should be done to investigate the efficacy of amantadine augmentation for TRD [89].

One limitation of this review is the lack of a standard definition of TRD. The discrepancies between how many antidepressants fail before depression is defined as “treatment-resistant” can vary from one to eight [4]. This causes difficulty when comparing the efficacy of different medications, since some studies may recruit participants who are more treatment-resistant than the participants in other studies. Differences in efficacy may be due in part to these discrepancies rather than the actual effects of the medication. Another limitation is the variation in rating scales. Some of these studies use MADRS, some use HAM-D, some use QIDS-SR, one uses CGI-I, and one uses BDI-II. It is possible that some of these scales differ in sensitivity. This could affect the accuracy of comparing the efficacy of different medications. However, a double-blind trial by Khan et al. found that MADRS is as sensitive as HAM-D for detecting antidepressant efficacy [90]. Another limitation is the file drawer effect, a theory that states that research without significant results is less likely to be published. Based on this, we may not know how many randomized control trials (RCTs) showed insignificant results for these drugs. Without this information, it is difficult to know for sure which medications are truly most effective. We attempted to limit the file drawer effect by following PRISMA guidelines.

With such a high percentage of patients failing to respond adequately to the first antidepressant they take, there is a need for standardized, evidence-based guidelines regarding first-line treatment strategies for TRD. Further investigation must be done to find a correlation between efficacy and the antidepressant that is being augmented. Most of the studies analyzed in this review do not standardize the antidepressants that the participants are taking. In these studies, some participants may take SSRIs, while others take tricyclic antidepressants, monoamine oxidase inhibitor (MAOIs), etc. These drugs have different mechanisms of actions, which could alter the efficacy of the augmentation agent it is paired with. Although some of these studies state that there was no significant correlation between drug class and efficacy, when groups were created based on drug class, many had a small sample size. Small sample sizes increase the margin of error in the study. It is possible that a correlation may be found if trials with more participants, receiving each type of antidepressant, are conducted.

## 4. Materials and Methods

The inclusion criteria used for this review were that studies must be RCTs with a sample of patients with MDD over the age of 18 who had failed to respond to at least one antidepressant. To narrow the scope of this review, medications included were limited to antidepressants, SGAs, lithium, thyroid hormone, lamotrigine, ketamine, and esketamine, since these were the most commonly researched medications.

**Figure 1:**
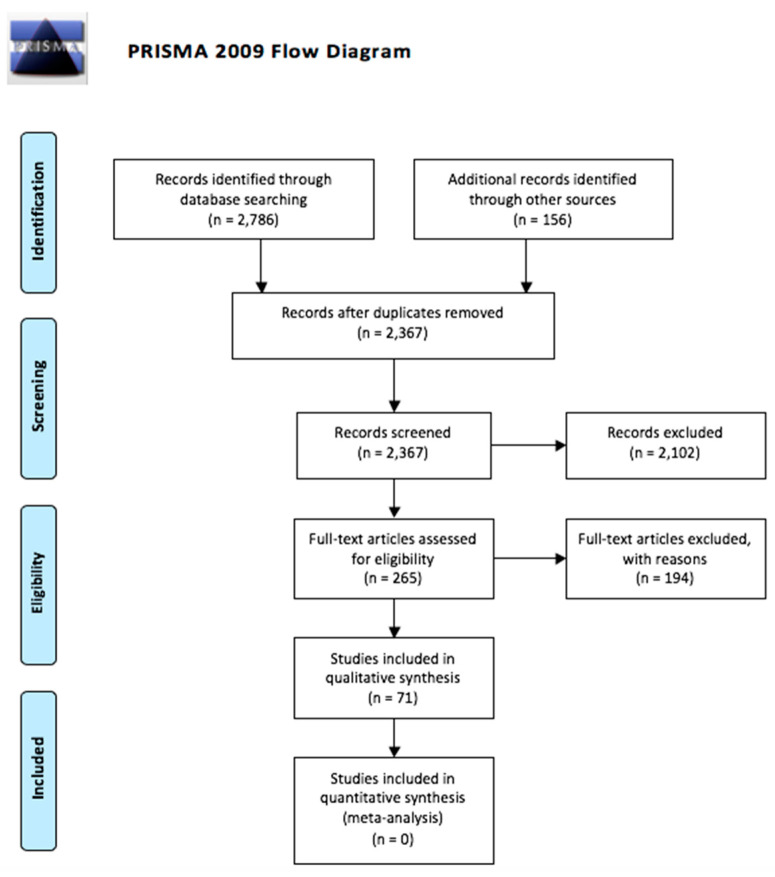
PRISMA flow diagram.

PRISMA guidelines were followed (Figure 1). Two electronic databases (PubMed and PsycINFO) were searched for publications, using the search terms “’refractory depression’ OR ‘TRD’ OR ‘resistant depression’ AND (augment* OR combin* OR switch*).” There were no search restrictions placed on publication year or type. 2786 articles resulted from this search. An additional 156 articles were retrieved from the bibliographies of relevant articles found from the initial PubMed and PsycINFO searches. 2367 articles remained after duplicates were removed. These 2367 articles were screened. 2102 articles were excluded, resulting in 265 articles remaining. These articles were read in full.

Of the 265 articles read in full, 71 articles were included in this qualitative synthesis. The list of 265 articles included a mixture of clinical trials, systematic reviews, meta-analyses, and case-studies and clinical trials. Only RCTs were included in the qualitative synthesis. All other types of articles were excluded. Next, RCTs that included children and adolescents were excluded because it is possible that these medications work differently in children than adults. Therefore, excluding studies that include children makes comparisons between different studies more accurate. The age range of the included studies is 18 to 80. From the remaining articles, we chose to only include articles that investigate the efficacy of medications from certain drug classes: SSRIs, SNRIs, atypical antidepressants, TCAs, MAOIs, SGAs, lithium, thyroid hormones, and anticonvulsants. Additionally, this article includes research on combining and switching antidepressants. The choice to focus on these medications was based on the relative amount and quality of articles found from the initial search. The included articles use varying definitions of TRD. It is important to note that some studies include partial responders, patients who experienced a symptom reduction greater than 25% but less than 50%. To address this variability, we will state the definition of TRD used in each study.

## 5. Conclusions

In conclusion, ketamine and esketamine appear to be effective medications for TRD. Specific SGAs, such as augmentation agents, are also effective for TRD. Lithium, T3, lamotrigine, and combining antidepressants, such as bupropion, mirtazapine, and TCAs, are also effective treatments for TRD. There is not enough research to conclude the efficacy of ziprasidone or T4. Very little comparative efficacy data between agents for TRD is available to inform treatment decisions. Clinicians should take a shared decision-making approach to take each patient’s predispositions and individual needs into consideration when choosing an antidepressant or augmentation agent.

## Figures and Tables

**Table 1:** Pharmacological Classes of Antidepressants.

Pharmacological Class	Neuroscience Based Class	Primary Target(s) ^1^	Examples
SSRI	5HT reuptake inhibitor	SERT	Citalopram, Sertraline
SSRI-‘plus’	5HT multimodal modulator	SERT and 5HT receptors	Vortioxetine
SNRI	5HT/NE reuptake inhibitor	SERT and NET	Venlafaxine, Duloxetine
TCA	NE reuptake inhibitor	SERT and NET	Imipramine, Desimpramine
MAOI	5HT, NE, DA multimodal modulator	MAO enzyme	Tranylcypromine
Atypical	DA/NE modulator	DAT and NET	Bupropion
Glutamate	Glutamate antagonist	NMDA	Esketamine, Ketamine

^1^ Information taken from Neuroscience Based Nomenclature [9]. SSRI = selective serotonin reuptake inhibitor; 5HT = 5 hydroxytryptophan; SNRI = serotonin-norepinephrine reuptake inhibitor; NE = norepinephrine; TCA = tricyclic antidepressant; MAOI = monoamine oxidase inhibitor; DA = dopamine.

**Table 2:** Pharmacological Classes of Augmentation Agents for Depression.

Pharmacological Class	Neuroscience Based Class	Primary Target(s) ^1^	Examples
Mood stabilizers	Lithium enzyme modulator	Phosphatidyl-inositol pathway ^2^	Lithium
SGA	DA/5HT antagonist, other	D_2_, 5HT_1_A, 5HT_2_A	Aripiprazole, Quetiapine
Thyroid	N/A	Thyroid hormone receptor	Liothyronine, Levothyroxine
Anticonvulsants	Glutamate channel blocker, other	Voltage-gated sodium channels, other	Lamotrigine, Valproate
Other	5HT partial agonist	5HT_1_A	Buspirone

^1^ Information taken from Neuroscience Based Nomenclature [9]. ^2^ Information taken from Brown et al. [10]; SGA = second generation antipsychotic.

**Table 3:** List of Included Antidepressant Studies.

Trial	Treatment Comparators
Lam et al., 2004 [16]	Bupropion SR; Citalopram; Bupropion SR plus Citalopram
Rush et al., 2006 [11]	Venlafaxine XR; Sertraline; Bupropion
Trivedi et al., 2006 [17]	Bupropion plus Citalopram; Buspirone plus Citalopram
Rosso et al., 2012 [23]	Duloxetine; Bupropion XR
Fornaro et al., 2014 [24]	Duloxetine; Bupropion; Duloxetine plus placebo
Mohammed et al., 2017 [15]	Aripiprazole plus antidepressant; Bupropion plus antidepressant; Bupropion
Fava et al., 1994 [25]	Fluoxetine; Fluoxetine plus Desipramine; Fluoxetine plus Lithium
Fava et al., 2002 [26]	Fluoxetine; Fluoxetine plus Lithium; Fluoxetine plus Desipramine
Nelson et al., 2004 [18]	Fluoxetine; Desipramine; Fluoxetine plus Desipramine
Thase et al., 2002 [12]	Imipramine; Sertraline
Carpenter et al., 2002 [21]	Mirtazapine plus antidepressant; placebo plus antidepressant
Fava et al., 2006 [13]	Mirtazapine; Nortriptyline
McGrath et al., 2006 [19]	Tranylcypromine; Venlafaxine plus Mirtazapine
Fang et al., 2010 [14]	Mirtazapine; Venlafaxine; Paroxetine
Kessler et al., 2018 [22]	Mirtazapine plus antidepressant; placebo plus antidepressant
Navarro et al., 2019 [20]	Mirtazapine plus Venlafaxine; Imipramine

SR = standard release; XR = extended release.

**Table 4:** List of Included Second Generation Antipsychotic Studies.

Trial	Treatment Comparators
Marcus et al., 2008 [27]	Aripiprazole plus antidepressant; placebo plus antidepressant
Berman et al., 2009 [29]	Aripiprazole plus antidepressant; placebo plus antidepressant
Yoshimura et al., 2012 [30]	Aripiprazole plus Paroxetine; Aripiprazole plus Sertraline
Fava et al., 2012 [31]	Aripiprazole plus antidepressant; placebo plus antidepressant
Yoshimura et al., 2014 [52]	Lithium plus Paroxetine; Olanzapine plus Paroxetine; Aripiprazole plus Paroxetine
Han et al., 2015 [28]	Aripiprazole plus antidepressant; switching antidepressants
Mohammed et al., 2017 [15]	Aripiprazole plus antidepressant; Bupropion plus antidepressant; switching to Bupropion
Dorée et al., 2007 [53]	Quetiapine plus antidepressant; Lithium plus antidepressant
Bauer et al., 2009 [40]	Quetiapine XR plus antidepressant; placebo plus antidepressant
Cutler et al., 2009 [38]	Quetiapine XR; Duloxetine
El-Khalili et al., 2010 [39]	Quetiapine XR plus antidepressant; placebo plus antidepressant
Bauer et al., 2013 [54]	Quetiapine XR plus antidepressant; Quetiapine XR; lithium plus antidepressant
Hobart et al., 2018 [41]	Quetiapine plus antidepressant; Brexpiprazole plus antidepressant; placebo plus antidepressant
Mahmoud et al., 2007 [47]	Risperidone plus antidepressant; placebo plus antidepressant
Reeves et al., 2008 [46]	Risperidone plus antidepressant; placebo plus antidepressant
Keitner et al., 2009 [48]	Risperidone plus antidepressant; placebo plus antidepressant
Fang et al., 2011 [49]	Valproate plus Paroxetine; Risperidone plus Paroxetine; Buspirone plus Paroxetine; Trazodone plus Paroxetine; Thyroid Hormone plus Paroxetine
Thase et al., 2015 [43]	Brexpiprazole plus antidepressant; placebo plus antidepressant
Thase et al., 2015 [44]	Brexpiprazole plus antidepressant; placebo plus antidepressant
Shelton et al., 2001 [33]	Olanzapine plus Fluoxetine; Olanzapine plus placebo; Fluoxetine plus placebo
Shelton et al., 2005 [35]	Olanzapine plus Fluoxetine; Olanzapine; Fluoxetine; Nortriptyline
Corya et al., 2006 [36]	Olanzapine plus Fluoxetine; Olanzapine; Fluoxetine; Venlafaxine
Thase et al., 2007 [34]	Olanzapine plus Fluoxetine; Fluoxetine; Olanzapine
Papakostas et al., 2012 [51]	Ziprasidone; placebo
Papakostas et al., 2015 [50]	Ziprasidone plus Escitalopram; placebo plus Escitalopram

XR = extended release.

**Table 5:** List of Included Lithium Studies.

Trial	Treatment Comparators
Heninger et al., 1983 [55]	Lithium plus antidepressant; placebo plus antidepressant
Zusky et al., 1988 [59]	Lithium plus antidepressant; placebo plus antidepressant
Schöpf et al., 1989 [56]	Lithium plus TCA; placebo plus TCA
Stein et al., 1993 [60]	Lithium plus TCA; placebo plus TCA
Joffe et al., 1993 [62]	Lithium plus TCA; T3 plus TCA; placebo plus TCA
Fava et al., 1994 [25]	Fluoxetine; Fluoxetine plus Desipramine; Fluoxetine plus Lithium
Katona et al., 1995 [57]	Lithium plus antidepressant; placebo plus antidepressant
Cappiello et al., 1998 [58]	Lithium plus Desipramine; placebo plus Desipramine
Fava et al., 2002 [26]	Fluoxetine; Fluoxetine plus Lithium; Fluoxetine plus Desipramine
Nierenberg et al., 2003 [61]	Lithium plus Nortriptyline; placebo plus Nortriptyline
Nierenberg et al., 2006 [63]	T3 plus antidepressant; Lithium plus antidepressant
Joffe et al., 2006 [64]	T3 plus Lithium plus antidepressant; T3 plus antidepressant; Lithium plus antidepressant; placebo plus antidepressant
Schindler et al., 2007 [65]	Lamotrigine plus antidepressant; lithium plus antidepressant
Bauer et al., 2013 [54]	Quetiapine XR plus antidepressant; Quetiapine XR; lithium plus antidepressant
Yoshimura et al., 2014 [52]	Lithium plus Paroxetine; Olanzapine plus Paroxetine; Aripiprazole plus Paroxetine

TCA = tricyclic antidepressant; T3 = triiodothyronine; XR = extended release.

**Table 6:** List of Included Thyroid Hormone Studies.

Trial	Treatment Comparators
Gitlin et al., 1987 [68]	T3 plus Imipramine; placebo plus Imipramine
Joffe et al., 1990 [66]	T3 plus TCA; T4 plus TCA
Joffe et al., 1993 [62]	T3 plus TCA; Lithium plus TCA; placebo plus TCA
Appelhof et al., 2004 [67]	T3 plus Paroxetine; placebo plus Paroxetine
Nierenberg et al., 2006 [63]	T3 plus antidepressant; Lithium plus antidepressant
Joffe et al., 2006 [64]	T3 plus Lithium plus antidepressant; T3 plus antidepressant; Lithium plus antidepressant; placebo plus antidepressant
Fang et al., 2011 [49]	Valproate plus Paroxetine; Risperidone plus Paroxetine; Buspirone plus Paroxetine; Trazodone plus Paroxetine; Thyroid Hormone plus Paroxetine

T3 = triiodothyronine; TCA = tricyclic antidepressant; T4 = thyroxine.

**Table 7:** List of Included Lamotrigine Studies.

Trial	Treatment Comparators
Normann et al., 2002 [72]	Lamotrigine plus Paroxetine; placebo plus Paroxetine
Barbosa et al., 2003 [71]	Lamotrigine plus Fluoxetine; placebo plus Fluoxetine
Schindler et al., 2007 [65]	Lamotrigine plus antidepressant; lithium plus antidepressant
Santos et al., 2008 [69]	Lamotrigine plus antidepressant; placebo plus antidepressant
Barbee et al., 2011 [70]	Lamotrigine plus Paroxetine; placebo plus Paroxetine

**Table 8:** List of Included Ketamine and Esketamine Studies.

Trial	Treatment Comparators
Berman et al., 2000 [73]	Ketamine; placebo
Zarate et al., 2006 [74]	Ketamine; placebo
Murrough et al., 2013 [75]	Ketamine; Midazolam
Lapidus et al., 2014 [81]	Ketamine; placebo
Singh et al., 2016 [82]	Esketamine; placebo
Lenze et al., 2016 [79]	Ketamine plus Clonidine
Su et al., 2017 [77]	Ketamine; placebo
Gálvez et al., 2018 [88]	Ketamine; Midazolam
Daly et al., 2018 [85]	Esketamine; placebo
Fava et al., 2018 [78]	Ketamine; Midazolam
Ionescu et al., 2019 [76]	Ketamine; placebo
Popova et al., 2019 [83]	Esketamine; placebo
Fedgchin et al., 2019 [84]	Esketamine; placebo
Domany et al., 2019 [80]	Ketamine; placebo
